# LumiraDX SARS-CoV-2 Antigen Test for Diagnosing Acute SARS-CoV-2 Infection: Critical Literature Review and Meta-Analysis

**DOI:** 10.3390/diagnostics12040947

**Published:** 2022-04-11

**Authors:** Giuseppe Lippi, Brandon M. Henry, Mario Plebani

**Affiliations:** 1 Section of Clinical Biochemistry and School of Medicine, University of Verona, 37134 Verona, Italy; 2 Cardiac Intensive Care Unit, The Heart Institute, Cincinnati Children’s Hospital Medical Center, Cincinnati, OH 45229, USA; brandon.henry@cchmc.org; 3 Disease Intervention & Prevention and Population Health Programs, Texas Biomedical Research Institute, San Antonio, TX 78245, USA; 4 Department of Medicine—DIMED, University of Padova, 35100 Padova, Italy; mario.plebani@unipd.it

**Keywords:** SARS-CoV-2, COVID-19, immunoassay, diagnosis, antigen

## Abstract

We present here a critical literature review and meta-analysis on the accuracy of the LumiraDX SARS-CoV-2 Antigen Test for diagnosing acute SARS-CoV-2 infection. An electronic search was conducted in the Scopus and Medline databases using the keywords “LumiraDX” AND “COVID-19” OR “SARS-CoV-2”, without date (i.e., up to 1 February 2022) or language restrictions, for detecting clinical studies where the diagnostic accuracy of the LumiraDX SARS-CoV-2 Antigen Test was compared with reference molecular diagnostic methods. All studies where the rates of true positive, true negative, false positive and false negative cases were available for constructing a 2 × 2 table and providing pooled estimates of diagnostic sensitivity, specificity and accuracy were included in a pooled analysis. The study was conducted in accordance with the PRISMA (preferred reporting items for systematic reviews and meta-analyses) reporting checklist. Eleven studies (*n* = 8527 samples) could be included in our pooled analysis, while five additional documents provided diagnostic accuracy data but could not be extracted for construction of a 2 × 2 table. The pooled diagnostic sensitivity and specificity were 0.86 (95%CI, 0.84–0.88) and 0.99 (95%CI, 0.98–0.99), respectively, while the area under the summary receiver operating characteristic curve was 0.974 (95%CI, 0.965–0.983) and the agreement was 96.8% (95%CI, 96.4–97.1%), with kappa statistics of 0.87 (95%CI, 0.85–0.88). In conclusion, the diagnostic performance of the LumiraDX SARS-CoV-2 Antigen Test would allow the conclusion that it may be seen as a reliable alternative to molecular testing for the rapid preliminary screening of acute SARS-CoV-2 infections.

## 1. Introduction

The dramatic and almost unpredictable clinical, social and economic burden caused by the ongoing coronavirus disease 2019 (COVID-19) pandemic is disrupting the efficiency of most healthcare systems worldwide [1], a situation that has recently become magnified by the continuous emergence of highly mutated lineages of the severe acute respiratory syndrome coronavirus 2 (SARS-CoV-2) [2]. Although the use of a nucleic acid amplification test (NAAT) aimed at detecting SARS-CoV-2 RNA in a diagnostic (preferably upper or lower respiratory tract) sample remains the gold standard for diagnosing an acute SARS-CoV-2 infection [3], the intrinsic characteristics of most molecular testing assays (i.e., low throughput, long turnaround time and the need for skilled personnel and dedicated laboratory instrumentation) prohibit providing a valid and timely result due to the enormous volume of tests that almost every laboratory is now facing, which is also associated with a paramount economic burden. This paves the way to the urgent need of identifying potential diagnostic alternatives suited for the purpose of combining high-volume and rapid testing.

An enormous number of rapid diagnostic tests aimed at detecting SARS-CoV-2 antigens (RDT-Ag) in upper respiratory tract specimens have been developed and commercialized (a detailed list can be found in the FIND database) [4]. Nonetheless, real-time evaluation and validation of many manual rapid immunoassays revealed a cumulatively low diagnostic accuracy, namely, an insufficient diagnostic sensitivity (i.e., around 70%) [5], which remains far below the minimum diagnostic sensitivity (i.e., ≥80%) required by the World Health Organization (WHO) [6] and the Task Force on COVID-19 of the International Federation of Clinical Chemistry and Laboratory Medicine (IFCC) [7]. Besides manual lateral flow (first- and second-generation) assays and laboratory-based chemiluminescent (fourth-generation) immunoassays [8], some intriguing and potentially valid alternatives are emerging, i.e., the so-called third-generation microfluidic assays, of which the LumiraDx SARS-CoV-2 Ag test represents the prototype for rapidity, handiness and potential availability as a decentralized testing device. Therefore, this article aims to present a critical literature review and meta-analysis of this innovative test in the diagnosis of acute SARS-CoV-2 infection. The following article is presented in accordance with the PRISMA (preferred reporting items for systematic reviews and meta-analyses) reporting checklist.

## 2. Materials and Methods

### 2.1. Immunoassay Description

The LumiraDx SARS-CoV-2 Ag Test (LumiraDx Ltd., Alloa, UK) is a microfluidic immunofluorescence assay for the direct and qualitative detection of SARS-CoV-2 antigens in nasal swab (NS) and nasopharyngeal swab (NPS) specimens from individuals with suspected COVID-19 or asymptomatic individuals. The test is meant to be used with the LumiraDx platform as a rapid point-of-care (POC) diagnostic assay. This particle-particle sandwich immunoassay is based on specific monoclonal antibodies coated on fluorescent latex nanoparticles and magnetic beads, which are directed against the SARS-CoV-2 nucleocapsid (N) protein. One drop of specimen collected within an extraction buffer is added to the reactive strip containing the dried reagents. The anti-SARS-CoV-2 antibodies coated on latex nanoparticles and magnetic beads react with the N antigen that is eventually present in the test sample to generate a sandwich immunocomplex. Microfluidic filtration eliminates free nanobeads but retains antigen-bridged immunocomplexes, which generate a fluorescent reaction whose intensity is proportional to the amount of analyte present in the sample. The results are then displayed on the touchscreen of the analyzer in less than 12 min. According to manufacturer’s specifications, the limit of detection (LoD) of this test is 32 median tissue culture infectious dose (TCID_50_)/mL and is linear up to 1.4 × 105 TCID_50_/mL. Both the test strips and analyzer contain quality control checks to ensure that the test is properly functioning.

### 2.2. Search Strategy

The search strategy used in this study is summarized in Table 1. Briefly, the electronic search was conducted in the two scientific databases Scopus and Medline (on the PubMed interface) based on the keywords “LumiraDX” and “COVID-19” or “SARS-CoV-2” within the search fields “TITLE” and “ABSTRACT” and “KEYWORDS”, with no date (i.e., up to 1 February 2022) or language restrictions, aimed at detecting potential documents that reported the diagnostic accuracy of the LumiraDX SARS-CoV-2 Antigen Test compared with reference molecular diagnostic methods. 

The two authors (G.L. and B.M.H.) assessed the title, abstract and full text (when available) of all items that could be detected based on the previously described search criteria, choosing clinical studies where the rates of true positive (TP), true negative (TN), false positive (FP) and false negative (FN) cases were available for constructing a 2 × 2 table. All references of these selected articles were also assessed for identifying other potentially includible studies. A pooled analysis based on the Mantel–Haenszel approach was finally conducted, aiming to estimate diagnostic sensitivity, specificity and accuracy (estimated as the summary receiver operating characteristic curve (SROC), agreement and Kappa statistics), with a 95% confidence interval (95%CI) and using a random effects model. Within study heterogeneity was calculated using the χ2 test and I^2^ statistic [9]. The statistical analysis was performed with Meta-DiSc 1.4 (Unit of Clinical Biostatistics team of the Ramón y Cajal Hospital, Madrid, Spain) [10]. 

This pooled analysis was conducted according to the preferred reporting items for systematic reviews and meta-analyses (PRISMA Checklist available as Supplementary File S1), in accordance with the Declaration of Helsinki and within the terms of local legislation. No ethical committee approval was necessary, as this is a critical literature review.

## 3. Results

The electronic search according to the predefined criteria allowed the identification of 26 publications once between-database duplicates were eliminated. Ten of these documents could not be included since no information on LumiraDx SARS-CoV-2 Ag Test for diagnosing acute SARS-CoV-2 infection was provided (*n* = 1), the article did not present diagnostic accuracy data (*n* = 2) or the document was a critical literature review (*n* = 4), a commentary (*n* = 2) or an erratum (*n* = 1). Five additional documents provided diagnostic accuracy data but not in such way to be included within a cumulative 2 × 2 table. Therefore, 11 studies (*n* = 8527 samples) could be included in our pooled analysis [11,12,13,14,15,16,17,18,19,20,21]. The main characteristics of these eleven studies are shown in Table 2.

Four studies were conducted in Italy, three in Germany, two in the US and one each in Spain and Senegal. Most studies used NPS (*n* = 7), while NS and oropharyngeal swabs (OPS) were used in five and one study (alone or in combination), respectively. The sample size ranged from 46 to 4146. Two studies were carried out in children and adult populations, four in adult populations and one study in only children, while in four studies the demographical characteristics of the population were not provided in the published resources (Table 2).

**Figure 1:**
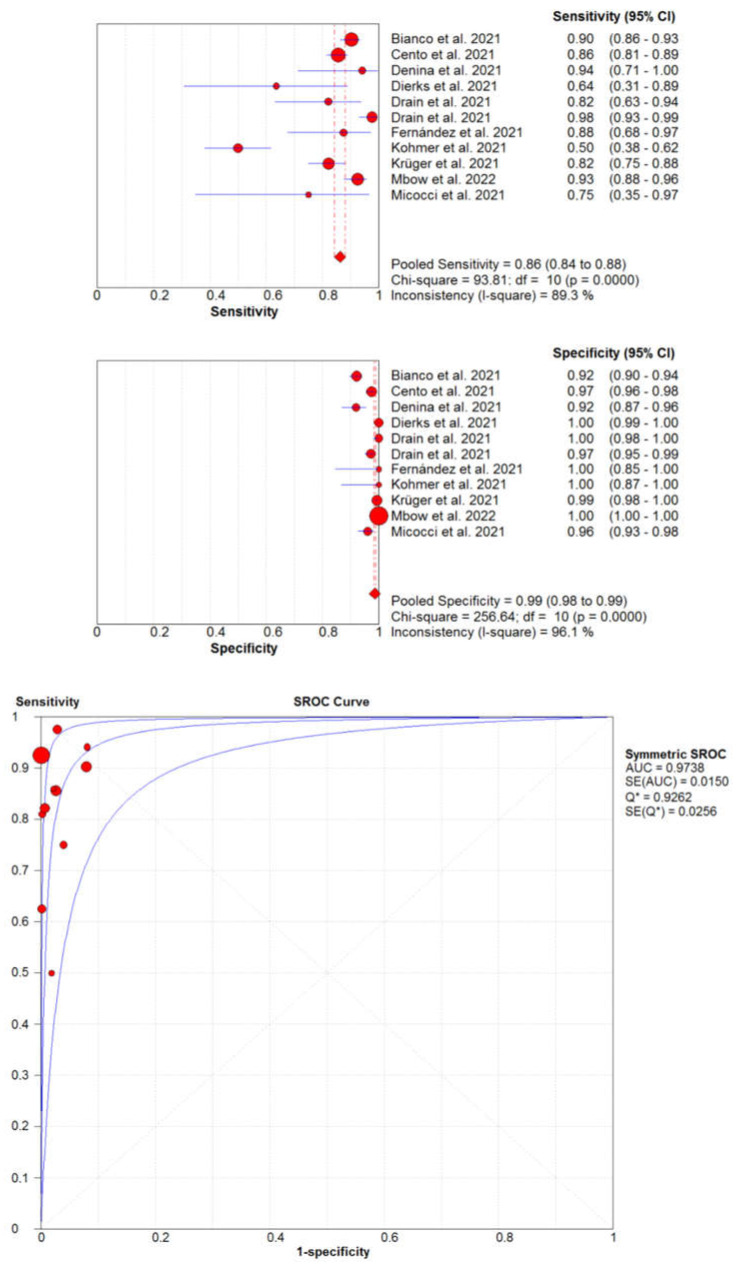
Pooled diagnostic performance of LumiraDX SARS-CoV-2 Antigen Test for diagnosing severe acute respiratory syndrome coronavirus 2 (SARS-CoV-2) infection. 95%CI, 95% confidence interval.

The diagnostic accuracy of the LumiraDx SARS-CoV-2 Ag Test compared to reference molecular biology assays is summarized in Figure 1. 

The pooled diagnostic sensitivity and specificity of this test were as high as 0.86 (95%CI, 0.84–0.88; I2, 89.3%) and 0.99 (95%CI, 0.98–0.99; I2, 96.1%), respectively, while its high diagnostic accuracy was mirrored by an area under the SROC of 0.974 (95%CI, 0.965–0.983), an accuracy of 96.8% (95%CI, 96.4–97.1%) and a kappa statistic of 0.87 (95%CI, 0.85–0.88), thus reflecting an almost perfect agreement with the reference NAATs [22]. Notably, although three studies accounted for over 70% of the samples, with one accounting for slightly less than 50%, their exclusion did not substantially modify the outcome of our analysis.

The description of the five studies [23,24,25,26,27] that did not provide sufficient information for constructing a 2 × 2 table is provided in Table 3 (sample size, n = 4073), showing that the cumulative diagnostic sensitivity ranged between 0.60–0.99 and the diagnostic specificity was between 0.99–1.00, respectively, thus closely mirroring the figures obtained in our pooled analysis (Figure 1). 

In the two studies that explored the concordance with molecular biology techniques, the agreement was as high as 96.3% and 96.9%.

Unfortunately, the data reported in the selected studies did not allow for the construction of a 2 × 2 table for samples with high viral load, and, hence, a sub-analysis of the diagnostic accuracy of the LumiraDX SARS-CoV-2 Antigen Test in those specimens could not be conducted. Nonetheless, the information on the diagnostic sensitivity in high-viral-load samples was provided in some of the studies, as follows: 0.91 (95%CI, 0.86–0.95) in NPS samples with Ct values ≤29 according to Cento et al. [12], 1.00 in both NS (95%CI, 0.94–1.00) and NPS (95%CI, 0.91–1.00) specimens with Ct values ≤33 according to Drain et al. [15], 0.93 (95%CI, 0.86–0.96) [15] in NPS samples with Ct values <25 according to Krüger et al. [19] and 0.92 (95%CI, 0.85–0.96) in OPS and NPS specimens with Ct values ≤33 according to Mbow et al. [20], while Kohmer et al. calculated a diagnostic sensitivity of 0.82 (95%CI, 0.66–0.93) in cell-culture-positive NPS samples [18].

## 4. Discussion

Due to the rapid and extreme surge in COVID-19 cases recorded all around the world connected to emergence of the new SARS-CoV-2 Omicron variant [28], which appears to be much more infective and resistant to natural and vaccine-induced immunity compared to the former lineages [29], the pressure on clinical laboratories has grown enormously to such a limit that many facilities are collapsing under the enormous volume of samples received [30]. This aspect has not only caused a substantial backlog of several days (or even weeks) for analyzing collected samples, but is also dramatically impairing the capacity to provide rapid test results for appropriate management of symptomatic COVID-19 cases, as well as for enabling reliable contact tracing with timely isolation of asymptomatic cases, which are still responsible for a substantial number of infections, especially those sustained by the new Omicron lineages [31].

In this extremely challenging and troublesome scenario, the use of rapid and accurate tests that may be able to support reference molecular assays for screening or even diagnosing acute SARS-CoV-2 infections appears to be the most suitable strategy. Although a kaleidoscope of RDT-Ags have been developed and commercialized so far, the vast majority of these do not reach such a sufficient level of diagnostic sensitivity to be used in routine clinical practice, with diagnostic sensitivities frequently below 50% [32,33,34]. Even those tests that would meet the criteria of minimum diagnostic sensitivity (i.e., ≥80%) [6,7] carry some additional drawbacks, such as being biased by arbitrary visual interpretation and providing mostly qualitative test results (which are hence unsuitable for longitudinal monitoring of viral load), along with the impossibility of being connected to the laboratory information system (LIS) for widespread and long-term data availability. In this perspective, the LumiraDx SARS-CoV-2 Ag Test represents a valuable opportunity, in that its diagnostic performance is aligned to those of the most sensitive SARS-CoV-2 antigen (lab-based) chemiluminescent immunoassays commercialized by DiaSorin, Roche, Ortho and Fujirebio (i.e., diagnostic sensitivity and specificity are comparable or even higher) [35,36,37,38], but it also comes as rapid POC instrumentation, thus enabling its usage outside the laboratory environment for purposes of mass (population) screening in various circumstances (e.g., crowded public places, schools, airports, social mass gatherings and so forth). Even within a hospital environment, the LumiraDx SARS-CoV-2 Ag Test may provide important benefits, such as rapid (i.e., within 12 min) patient screening in short-stay units (e.g., emergency room), longitudinal bedside monitoring of viral load in sub-intensive and intensive care wards or even widespread healthcare staff and patient testing for the rapid identification of infective clusters. Notably, the already optimal performance of the assay is likely magnified in upper respiratory tract specimens with high viral load, thus representing an ideal and versatile test for identifying the so-called “super-carriers” who, incidentally, are also “super-spreaders” of the virus [39].

## 5. Conclusions

The diagnostic performance of the LumiraDX SARS-CoV-2 Antigen Test would allow the conclusion that it may be considered a cost-effective, much handier alternative, with only a slightly less reliable outcome than molecular testing for rapid preliminary screening of acute SARS-CoV-2 infections, especially in clinical specimens bearing high viral loads. Notably, a careful Bayesian-oriented approach should always be carried out, discouraging the deployment of these tests in contexts where the prevalence of COVID-19 is low and there are no reported contacts with positive cases. In such cases, the use of molecular assays is still almost unavoidable.

## Figures and Tables

**Table 1:** The search strategy summary.

Items	Specification
Date of Search	1 February 2022
Databases and other sources searched	Scopus, Medline (PubMed interface)
Search terms used	“LumiraDX” AND “COVID-19” or “SARS-CoV-2”
Timeframe	Up to 1 February 2022
Inclusion and exclusion criteria	No date or language restrictions, clinical studies where the rates of true positive (TP), true negative (TN), false positive (FP) and false negative (FN) cases compared to reference SARS-CoV-2 molecular biology techniques were available for constructing a 2 × 2 table
Selection process	Conducted by G.L., verified by B.M.H.

**Table 2:** Summary of studies that investigated the cumulative diagnostic performance of LumiraDX SARS-CoV-2 Antigen Test for diagnosing severe acute respiratory syndrome coronavirus 2 (SARS-CoV-2) infection.

Study	Country	Sample Matrix	Sample Size (*n*)	Population	Molecular Assay (Gene Targets)
Bianco et al. 2021 [11]	Italy	NS and NPS	907	Median age 48 (range, 0.2–94) years; 56% females	Cepheid Xpert Xpress SARS-CoV-2 PCR
Cento et al. 2021 [12]	Italy	NPS	959	Median age 66 (IQR, 45–79) years; 42.2% females	In-house
Denina et al. 2021 [13]	Italy	NPS	191	Median age 5.8 (IQR, 1.1–10.8) years, 46% females	Diasorin Simplexa COVID-19 Direct kit
Dierks et al. 2021 [14]	Germany	NPS	444	N/A	Primerdesign Genesig Real-Time PCR Coronavirus (COVID-19) assay, Cepheid Xpert Xpress SARS-CoV-2 PCR and Roche Cobas 6800 SARS-CoV-2 Test
Drain et al. 2021 [15]	USA	NS	222	Mean age, 39 ± 17 years; 63% females	Roche Cobas 6800 SARS-CoV-2 Test and Thermo Fisher TruGenX
Drain et al. 2021 [16]	USA	NS and NPS	512	Mean age, 34 ± 19 years; 56% females	Roche Cobas 6800 SARS-CoV-2 Test
Fernández et al. 2021 [17]	Spain	NS and NPS	46	N/A	Seegene Allplex SARS-CoV-2 assay
Kohmer et al. 2021 [18]	Germany	NPS	100	N/A	Roche Cobas 6800 SARS-CoV-2 Test
Krüger et al. 2021 [19]	Germany	NPS	761	Median, 35 (IQR, 27–42) years; 52% females	Seegene Allplex SARS-CoV-2 assay and Roche Cobas 6800 SARS-CoV-2 Test
Mbow et al. 2022 [20]	Senegal	OPS and NPS	4146	Age range, 2–96 years; 47% females	Seegene Allplex SARS-CoV-2 assay
Micocci et al. 2021 [21]	Italy	NS	239	N/A	N/A

IQR, interquartile range; N/A, not available; NPS, nasopharyngeal swab; NS, Nasal swab; OPS, oropharyngeal swab; S/C, signal/cutoff ratio.

**Table 3:** Summary of additional studies that investigated the diagnostic performance of LumiraDX SARS-CoV-2 Antigen Test for diagnosing severe acute respiratory syndrome coronavirus 2 (SARS-CoV-2) infection but could not be included in the pooled analysis.

Study	Country	Sample Matrix	Sample Size	Sensitivity	Specificity	Accuracy
Burdino et al. 2021 [23]	Italy	NS and NPS	1232	0.90 (95%CI, 0.86–0.93)	0.99 (95%CI, 0.99–1.00)	Concordance: 96.9%
Gresh et al. 2021 [24]	USA	NS	2241	N/A	1.00 (95%CI, 0.99–1.00)	Agreement: 96.3%
Greub et al. 2021 [25]	Switzerland	NPS	200	0.99 (95%CI, 0.93–1.00)	0.99 (95%CI, 0.99–1.00)	N/A
Karon et al. 2021 [26]	USA	NPS	350	0.83 (95%CI, 0.77–0.88)	1.00 (95%CI, 0.98–1.00)	N/A
Scheiblauer et al. 2021 [27]	Germany	OPS and NPS	50	0.60 (95%CI, N/A)	N/A	N/A

N/A, Not available, NS, nasal swab; NPS, nasopharyngeal swab; OPS, oropharyngeal swab.

## Data Availability

Data will be available upon reasonable request to the corresponding author.

## Supplementary Materials

The following supporting information can be downloaded at: https://www.mdpi.com/article/10.3390/diagnostics12040947/s1, File S1: PRISMA Checklist.
